# The political undertones of building national health research systems – reflections from The Gambia

**DOI:** 10.1186/1478-4505-7-13

**Published:** 2009-05-29

**Authors:** Ayo Palmer, Samuel E Anya, Paul Bloch

**Affiliations:** 1 CIAM – Public Health Research & Development Centre, Kanifing Institutional Layout, Kanifing, The Gambia; 2 DBL – Centre for Health Research & Development, Faculty of Life Sciences, University of Copenhagen, Copenhagen, Denmark

## Abstract

In developing countries building national health research systems is a movement similar to a political leadership contest. Increasingly, political campaigns to select leaders depend less on ideologies and political messages and more on promising change that will promptly improve the quality of life of the voters. In this process the benefits and risks of every action and statement made by the candidates are carefully assessed.

Approaches currently promoted to strengthen health research within ministries of health in developing countries place emphasis on implementing logical steps towards building national health research systems including developing a national health research policy and strategic plan, conducting a situational analysis of research in the country, setting a national health research agenda, establishing research ethics and scientific committees, and building human and institutional capacity for health research management and conduct. Although these processes have successfully improved the standards of health research in some settings, many developing countries struggle to get the process going. One reason is that this approach does not deal with basic questions posed within a ministry of health, namely, "What is the political benefit of the ministry assuming control of the process?" and "What are the political implications for the ministry if another institution spearheads the process?"

Seen from the perspective of non-governmental organizations, academic institutions and donors trying to support the processes of strengthening national health research systems, one of the foremost activities that needs to be undertaken is to analyze the political context of national health research and, on that basis, plan and implement appropriate political health research advocacy initiatives. This includes the development of explicit messages on the political benefits to the leadership in the ministry of health of their role in the conduct, management and dissemination of health research within the country. Civil society organizations, with links to both government and non-governmental organizations, are well placed to play the role of advocates.

It is only through broad and active participation of stakeholders that the process of developing effective and sustainable national health research systems will truly become a national movement inspired, led and sustained by ministries of health.

## Background

The renewed interest in global issues related to research for health emphasizes the value and role of health research in health development [1,2]. This realization has given rise to a number of high level declarations and a Global Ministerial Call to Action to strengthen research for health, development and equity [3-5].

However, developing countries face several challenges in responding to the need for knowledge to inform decision-making. Collectively, these relate to the setting up of effective national health research systems. Specifically, these include lack of legal and strategic frameworks for research; lack of credible governance structures for research; lack of coordination of research activities; inadequate participation of stakeholders in research, policy and implementation processes; lack of demand for research; low accessibility and use of research findings; and inadequate financial and human capacity [6,7]. Another less well described challenge is how to incorporate political perspectives (views about authority and control of health research) that influence the effectiveness of a national health research system.

In developing countries building national research systems can be viewed as a movement similar to a political leadership contest. Increasingly, political campaigns to select leaders depend less on ideologies and more on promising to meet the felt needs of voters [8]. In this process voters carefully assess the personal benefits and risks of every action and statement made by the candidates.

Similarly, a national health research system (the political candidate) must go beyond a focus on inclusiveness, (semi)-independence, transparency and accountability [9] (ideologies) to address the unspoken concerns of stakeholders (the voters). The government, in most countries, is the most important voter and its explicit support is necessary to establish a robust national health research system.

The perspectives of a non-governmental organization are described based on its experiences of participating in the process of developing a National Health Research Policy as the political and legal foundation for strengthening the national health research system in The Gambia.

## Context of health research system development in The Gambia

In The Gambia, national public health programmes are grappling with how best to achieve high coverage rates to deal with the most pressing health problems faced by the population. The urgency and need to address an increasingly complex situation in the health sector still characterized by high burden of communicable diseases, a rising number of cases of injuries from car accidents and non-communicable diseases, limited health budgets and a human resource crisis has led to an increasing awareness and appreciation of the value of research to objectively address health systems and service delivery problems.

Most health research has been conducted using external resources by the Medical Research Council of The Gambia which is a non-government research institution. Some public sector institutions conduct research but these are usually small studies to identify and address operational issues. Studies published in international peer-reviewed scientific journals are usually those undertaken by the Medical Research Council. Although research activities have been going on in the country for several decades and governance structures such as scientific and ethics committees exist, there has been no legal or political framework for a national system.

In 2002 the Ministry of Health, with support from the African Development Bank and the World Health Organization (WHO), embarked on the process of developing a national health research system with the development of a National Health Research Policy as a first step. The need for a Policy was recognized by the Ministry of Health but the process depended on financial and technical support provided by Development Partners. The aim of the Policy is to coordinate and promote research on health problems of relevance to the health needs of the population. The Ministry appointed a core team to spearhead the process in partnership with a wide range of partners from public, private and nongovernmental sectors.

## Cooperation between partners in developing a National Health Research Policy

The main partners were the Ministry of Health, academia, civil society organizations, and development partners. Partners undertook tasks based on perception of needs they could meet. The main role played by each partner is summarized below.

### Ministry of Health

The Ministry of Health provided leadership for the process. The first draft of the policy was developed within the ministry with technical assistance provided by WHO. The ministry subsequently invited other stakeholders to contribute to the development of the policy, communicated and corresponded with partners, chaired meetings, made presentations on the contents of the health policy, responded to questions, discussed with donors, formally briefed health leadership, and drafted a Cabinet paper on the National Health Research Policy.

### Civil Society Organizations

Civil Society Organizations (CSO) provided support for logistics, acted as the secretariat, conducted background research and situational analyses, edited contents of the policy, and conducted advocacy with health leadership.

### Academia

These were represented by the University of The Gambia, Medical Research Council and Gambia College. They provided technical guidance on models of governance and on the type and range of technical competencies needed to set up a national health research system.

### Development partners

The main partners were WHO and UNICEF. They provided financial and technical support as well as perspectives on the rationale and attractiveness of setting up a robust governance structure to coordinate and provide oversight of health research activities in the country.

During the development of the National Health Research Policy, a number of related initiatives were undertaken in the period from 2002–2008, namely 1) the implementation of a national review of ethics in health research, 2) the establishment of an Ethics Committee at the National Teaching Hospital, and 3) the establishment of a Research & Publications Committee at the University of The Gambia.

The second draft of the Policy was completed in 2007. This was followed by advocacy efforts undertaken by the relevant technical unit in the Ministry and CSO representatives. The technical unit formally submitted the draft policy document and briefed the upper hierarchy of the Ministry. The advocacy undertaken by CSO representatives concentrated on several subjects: raising awareness on the importance of effective coordination of research activities by streamlining governance structures; extending scientific scrutiny to operational research activities; and the uniqueness within the context of the country of setting up a single national governance structure to coordinate research activities.

Despite advocacy efforts, however, and the inclusive consultative process initiated and coordinated by the relevant technical unit of the Ministry, the process of submitting the National Health Research Policy for Cabinet approval stalled. This was due to perceived unspoken concerns that ownership of the national health research system may end up outside the public sector; concerns originating from perceived imbalances in research capacity between the public sector and the more highly capacitized non-government research institutions.

For this reason the focus of advocacy efforts changed to emphasize how public sector leadership of the proposed governance structure can assure broad ownership of the national health research system.

The experiences in this process have generated valuable lessons that can contribute to the on-going debate on the political dimension of how to strengthen national health research systems in low income countries.

## Lessons learned

1. In retrospect, the assumption that an appreciation of the benefits of the Policy would be shared by all interest groups within the Ministry of Health was shortsighted. All parties were clear on the potential technical benefits of developing a National Health Research Policy as a first step towards strengthening the national health research system in The Gambia. These included setting the stage for developing research priorities based on real needs of the health sector, improving accessibility of data/information for decision making and the use of data for resource mobilization. What was less apparent was the perspective of the upper hierarchy in the Ministry for whom questions of power related to ownership and control of health research were foremost.

The *political *interests of promoting a national health research system in The Gambia, and presumably in other developing countries, include:

• Ensuring that research is internally driven, owned by the country and focused on national priorities

• Ensuring that research can be used to identify health problems and service delivery bottlenecks of immediate concern to the country.

• Ensuring that research feeds into policy and practice to protect and improve health of the population in the context of national development.

2. In Ministries of Health where the political and technical leadership differ in their perspectives on health research both technical and political benefits have to be clear. Understanding these perspectives by gathering views and opinions on technical and political perspectives on health research is necessary for fostering broad participation and ownership of the process of developing health research policies and systems.

3. Leadership in the Ministry of Health is critical for the development of health research policies and systems. Such processes must be spearheaded by dedicated high-level authorities who acknowledge the value of a national health research system and who are prepared to take advantage of emerging opportunities (financial and technical) to set it up. These processes require designated and long-term financial and technical resources to prevent untimely interruptions or termination. Commitments made in Declarations on Health Research also contribute to sustaining interest in developing policies and systems.

4. For government to support and facilitate the establishment of a robust national health research policy and system, deep-seated but often unexpressed concerns about ownership and control of the research process need to be identified and addressed.

Key concerns relate to:

• The need for strengthened national regulation around ethical approval and conduct of research

• The need to channel research funds into research aiming at strengthening the health system

• The need to identify ways of using research findings, including intellectual property, to the direct benefit of the country

5. Resistance to a health research policy or reluctance to support it is not necessarily a negative sign but may be an indication of government's commitment to ensuring that the policy is one it can "buy in to". Supporting the policy without critical reflection and comment by the health leadership may be indicative of disinterestedness that would ultimately undermine adherence to the policy, effectiveness of a health research system and the use of research findings for policy change and action.

6. In The Gambia, the felt need is for research to improve health and health systems. In such a situation, the importance of other relevant ministries such as finance, higher education, environment and science and technology may be overlooked. On one hand, the Ministry of Health may not immediately see the value of engaging the other ministries. On the other hand, the other ministries may see health research as the sole business of the health ministry. The lack of engagement may be, in reality, one of the consequences of the underlying dispositions about ownership and control of health research.

7. Civil Society Organizations (CSOs) engaged in health research can play multiple complementary roles in the process of developing health research policies and systems [10]. They have the flexibility to engage and advocate at different levels within the Ministry, a role that staff within the Ministry cannot easily undertake because of institutional hierarchy. This engagement provides an understanding of the differing political perspectives and helps to position health research in a way that is politically appealing. Furthermore, speaking from a position of active involvement in health research allows one to be credible when advocating in the Ministry. On the other hand, there are a number of risks. The credibility and effectiveness of CSOs may be compromised if they are perceived as pushing their own agenda and/or as trying to usurp the role of the Ministry or technical units within the Ministry. Thus, the CSOs need to be seen and perceived as championing the interests of the country and the Ministry and not their own interests or those of external institutions. This requires sensitivity, tact and diplomacy that would depend on the particular context.

## Research Priorities

Research priorities emanating from this case study are as follows:

• What factors promote ownership of national health research systems by governments in low-income countries including those related to governance, leadership and stewardship of the research process and organization and governance of the research system?

• How can the concerns of the political leadership over the governance of health research be identified early and systematically?

• How can the political dimensions of health research in developing countries be used to develop advocacy strategies to engage the political leadership?

• What can be done to promote the adoption of best practices and known interventions that improve the effectiveness of processes that have been used to set up national health research systems?

## Policy Implications

The following policy recommendations are made within the context of strengthening government's ownership and control of the national research process.

1. In the process of setting up a national health research system, policy makers in the health ministry and related ministries such as the Ministry of Finance, Higher Education and Science and Technology should be consulted early to gather their perspectives on health research and encourage their participation in the process.

2. The health ministry should have a leadership role within the proposed governance structure set up to oversee health research activities to foster shared/broad ownership of the research process.

3. Advocacy efforts on both technical and political benefits of a health research policy should be directed at policy makers when setting up a national health research system.

## Conclusion

The research issues and policy implications identified in this paper provide an important contribution to current global efforts to support active involvement of national governments in health research activities. The experience described points to the importance of understanding perceptions and processes that go beyond the logical paradigm currently promoted for the establishments of health research systems within countries.

The key challenge is to develop systematic mechanisms that will reveal the innermost feelings, pre-conceived notions and values of all stakeholders about health research and the proposed health research system. Such feelings, notions and values are deep-rooted and subconscious but will determine behaviour and involvement in the research system. In political campaigns, good polling is used in an attempt to characterize the sacrosanct values that voters attach to specific issues [11,12]. The findings of such polling are then used to develop personalized communication messages that address the deep-seated concerns of the voters and encourage them to accept the Candidate [8,11].

To ensure there are no surprises during the development and implementation of national health research systems (election day), we need to develop and deploy approaches that address the "unsaid".

## Competing interests

The authors declare that they have no competing interests.

## Authors' contributions

AP conceived the paper and wrote the first draft. SEA and PB contributed to drafting the manuscript. All authors participated in the policy development process.
